# Endosomal H_2_O_2_ Molecules Act as Signaling Mediators in Akt/PKB Activation

**DOI:** 10.3390/antiox14050594

**Published:** 2025-05-16

**Authors:** Sujin Park, Chaewon Kim, Sukyeong Heo, Dongmin Kang

**Affiliations:** 1 Department of Life Science, Fluorescence Core Imaging Center and Bioimaging Data Curation Center, Ewha Womans University, Seoul 03760, Republic of Korea; sujin.park27@gmail.com (S.P.); kcw4269@naver.com (C.K.); hcjm123@gmail.com (S.H.); 2 Department of Biomedical Engineering, Dongguk University, Seoul 10326, Republic of Korea

**Keywords:** receptor-mediated endocytosis, hydrogen peroxide, Akt/PKB, early endosome, the Leucine zipper motif 1 (APPL1)

## Abstract

Receptor-mediated endocytosis (RME) is a commonly recognized receptor internalization process of receptor degradation or recycling. However, recent studies have supported that RME is closely related to signal propagation and amplification from the plasma membrane to the cytosol. Few studies have elucidated the role of H_2_O_2_, a mild oxidant among reactive oxygen species (ROS) in RME and second messenger of signal propagation. In the present study, we investigated the regulatory function of H_2_O_2_ in early endosomes during signaling throughout receptor-mediated endocytosis. In mammalian cells with a physiological amount of H_2_O_2_ generated during epidermal growth factor (EGF) activation, fluorescence imaging showed that the levels of two activating phosphorylations on Ser^473^ and Thr^308^ of Akt were transiently increased in the plasma membrane, but the predominant p-Akt on Ser^473^ appeared in early endosomes. To examine the role of endosomal H_2_O_2_ molecules as signaling mediators of Akt activation in endosomes, we modulated endosomal H_2_O_2_ through the ectopic expression of an endosomal-targeting catalase (Cat-Endo). The forced removal of endosomal H_2_O_2_ inhibited the Akt phosphorylation on Ser^473^ but not on Thr^308^. The levels of mSIN and rictor, two components of mTORC2 that work as a kinase in Akt phosphorylation on Ser^473^, were also selectively diminished in the early endosomes of Cat-Endo-expressing cells. We also observed a decrease in the endosomal level of the adaptor protein containing the PH domain, the PTB domain, and the Leucine zipper motif 1 (APPL1) protein, which is an effector of Rab5 and key player in the assembly of signaling complexes regulating the Akt pathway in Cat-Endo-expressing cells compared with those in normal cells. Therefore, the H_2_O_2_-dependent recruitment of the APPL1 adaptor protein into endosomes was required for full Akt activation. We proposed that endosomal H_2_O_2_ is a promoter of Akt signaling.

## 1. Introduction

Hydrogen peroxide (H_2_O_2_) molecules are produced during aerobic respiration in mammalian cells; their generation is involved in diverse physiological events, including cell proliferation and signal transduction [1,2,3]. Because high H_2_O_2_ concentrations are toxic to cells, organisms have evolved to develop an efficient system consisting of abundant antioxidant proteins to remove H_2_O_2_ and locally control its generation to avoid the unwanted modification of macromolecules. Studies have revealed compelling evidence to support the physiological role of local H_2_O_2_ molecules as signaling mediators. For instance, localized H_2_O_2_ accumulation in mammalian cells is necessary to propagate receptor-mediated signaling in lipid rafts of the plasma membrane or redox-active endosomes (redoxosomes); it is also substantial for cell cycle progression via Cdk1 activation around the centrosome at the G_2_-M transition [4,5,6,7,8].

In terms of H_2_O_2_-mediated signaling cascades at the plasma membrane in cells activated by growth factors such as the epidermal growth factor (EGF), platelet-derived growth factor (PDGF), or insulin-like growth factor (IGF), further studies should elucidate the regulatory mechanism of the phosphoinositide 3-kinase (PI3K)-Akt/PKB-mTORC axis by H_2_O_2_ to understand the role of H_2_O_2_ in metabolic diseases and cancer progression. PI3K activated by growth factor stimulation produces PtdIns(3,4,5)P_3_ from PtdIns(4,5)P_2_ at the plasma membrane; thereafter, the SH2-containing inositol 5′-phosphatase (SHIP) generates PtdIns(3,4)P_2_ [9,10,11]. The pleckstrin homology (PH) at the amino terminus of Akt should directly bind to PtdIns(3,4,5)P_3_ and PtdIns(3,4)P_2_ to fully activate Akt Ser and Thr kinase, which is important for Akt to form local signaling complexes containing its upstream kinases, namely, phosphoinositide-dependent protein kinase 1 (PDK1) or the mechanistic target of rapamycin complex 2 (mTORC2) [12,13,14]. H_2_O_2_ accumulates locally at the plasma membrane in cells in response to stimulation by growth factors through the production of activated NADPH oxidase (Nox) and the inactivation of antioxidant protein peroxiredoxin (Prx) [7,8]. Phosphatase and tensin homolog (PTEN) lipid phosphatase, which dephosphorylates PtdIns(3,4,5)P_3_ to PtdIns(4,5)P_2_, acts as a primary terminator of PI3K-Akt signaling. PTEN deficiency commonly occurs in patients with cancer; it is related to the hyperactivation of Akt signaling. When the levels of H_2_O_2_ molecules increase in response to growth factor activation, PTEN 3-phosphatase becomes inactivated through the oxidation of the catalytic Cys^124^ residue of PTEN, and PtdIns(3,4,5)P_3_ accumulates, which is critical for Akt signaling [15,16]. PTEN is a major target of H_2_O_2_-dependent oxidation in Akt activation, considering that Nox1-overexpressing cells in response to growth factor activation show a higher PtdIns(3,4,5)P_3_ level than control cells do, while Prx II-overexpressing cells have a lower PtdIns(3,4,5)P_3_ level than control cells [15]. Akt is activated by PtdIns(3,4,5)P_3_ mostly at the plasma membrane of cells activated by growth factors. When H_2_O_2_ accumulates in the plasma membrane, Akt is activated via the transient increase in PtdIns(3,4,5)P_3_ by H_2_O_2_-dependent PTEN inactivation.

In addition to Akt activation at the plasma membrane, the phosphorylation of several Akt substrates occurs on the endomembrane, which contains PtdIns(3,4)P_2_, a possible phosphoinositide for Akt recruitment [17]. Recently, we demonstrated that in PtdIns(3,4)P_2_-dependent endosomal Akt activation, the mTORC2 complex should be recruited to endosomal Akt [18]. This event is important for the Akt signaling pathway through GSK 3β and forkhead box O1/O3 (FOXO1/3) phosphorylation. Specifically, the phosphorylations of Ser^9^ of GSK 3β and Thr^1462^ of tuberous sclerosis 2 (TSC2) are dependent on endosome-associated Akt [19,20,21,22]. The endocytosis of receptors activated at the plasma membrane is regarded as a feedback inhibition mechanism of a cell to downregulate its signaling by degrading its surface receptors in an endo-lysosome-dependent pathway. However, increasing evidence indicates that the endocytic pathway is necessary to amplify and transduce signals from signaling complexes containing activated receptors [23,24,25]. One of the key adaptor proteins for endosomal Akt activation is APPL1 (Adaptor protein containing PH domain, PTB domain, and Leucine zipper motif), a Rab5 effector in early endosomes [22,26,27,28]. APPL1 proteins bind not only to Akt [29] but also to a number of activated receptors, such as TrkA [30,31] and adiponectin [32,33]. Previous studies proposed that the APPL protein can coordinate signal transduction in early endosomes because it binds to small GTPase Rab5-GTP, an endosomal protein [27]. Endosomal APPL1 protein is required for GSK 3β phosphorylation but not for TSC2 phosphorylation; therefore, Akt substrate specificity is controlled by endosome-associated Akt [28]. However, studies have yet to elucidate the effects of a local increase in H_2_O_2_ levels at the endomembrane on the regulation of Akt activity.

Here, we used an endosomal-targeting H_2_O_2_ sensing reporter to observe the increase in endosomal H_2_O_2_ levels during receptor-mediated endocytosis (RME) in cells activated by growth factors. We found that the local increase in H_2_O_2_ around early endosomes by activated Nox I was vital for Akt activation through the recruitment of APPL, an adaptor of Akt and mTORC2, a specific kinase; this increase was distinct from the transient increase in PtdIns(3,4,5)P_3_ levels by H_2_O_2_ at the plasma membrane.

## 2. Materials and Methods

### 2.1. Materials

The following substances were used: diphenylene iodonium (DPI, ALX-430-005-M005; Enzo Life Sciences, Farmingdale, NY, USA); epidermal growth factor (EGF, PHG0311; Invitrogen, Carlsbad, CA, USA); glucose oxidase (GOx, 345386; Sigma-Aldrich, St. Louis, MO, USA); 4′,6-diamidino-2-phenylindole (DAPI, 10236276001; Roche, Basel, Switzerland); Alexa-568 conjugated Transferrin (T23365) and Alexa-555 conjugated EGF (E35350; Thermo Fisher Scientific, Waltham, MA, USA); mouse monoclonal antibody to GFP (A11120) and rabbit polyclonal antibody to GFP (A11122; Thermo Fisher Scientific, Waltham, MA, USA); mouse monoclonal antibody to Rictor (ab56578; Abcam, Cambridge, MA, USA); mouse monoclonal antibody to mSIN1 (05-1044) and mouse monoclonal antibody to α-actin (AC-74, A3853; Sigma-Aldrich, St. Louis, MO, USA); rabbit polyclonal antibody to catalase (LF-PA0060; AbFrontier, Seoul, Republic of Korea); rabbit monoclonal antibody to Rab5 (3547), rabbit polyclonal antibody to phospho-Akt (Ser^473^; 9271), rabbit monoclonal antibody to phospho-Akt (Thr^308^; 13038), rabbit polyclonal antibody to Akt (9272), and rabbit monoclonal antibody to APPL1 (3858; Cell Signaling Technology, Danvers, MA, USA); and mouse monoclonal antibody to EEA1 (610457; BD Biosciences (Franklin Lakes, NJ, USA).

### 2.2. Cell Culture and Transfection

Cos7 and HeLa cells were cultured in high-glucose Dulbecco’s modified eagle medium (DMEM, LM001-05; Welgene, Gyeongsan, South Korea) supplemented with 10% (*v*/*v*) fetal bovine serum (FBS, Gibco, Grand Island, NY, USA) and 1% penicillin/streptomycin (Hyclone, Logan, UT, USA) at 37 °C in a humidified atmosphere containing 5% CO_2_. Transfections were performed using Effectene (Qiagen, Hilden, Germany) in accordance with the manufacturer’s protocol. Neon electroporation (Invitrogen, Carlsbad, CA, USA) was used following the manufacturer’s instructions to transfect mouse embryonic fibroblasts (MEFs) and introduce siRNAs to HeLa cells. For Cos7 cells to be exposed to a concentration of H_2_O_2_ constantly, GOx (20 mU/mL) was added into a high-glucose medium. For Cos7 and HeLa cells to be stimulated with growth factors, cells were deprived of serum for 5 h and then treated with EGF (200 ng/mL) at the selected time points. To reduce intracellular H_2_O_2_ levels, Cos7 cells were incubated in the presence of DPI (10 μM) for 30 min and incubated with additional EGF (200 ng/mL) at the selected time points. Catalase was administered into Cos7 cells as previously described [34]. The cells were incubated with catalase (2 mg/mL) in 3 mL of Lipofectamine solution (prepared with 60 μL of Lipofectamine 2000 [Invitrogen, 11668-019] in 1.5 mL of DMEM and 6 mg of catalase [Sigma-Aldrich, C1345] in 1.5 mL of DMEM for 20 min) at 37 °C for 5 h.

### 2.3. Plasmids and siRNAs

dsRNA oligos were synthesized by Dharmacon (Lafayette, CO, USA), and the target sequence of the siAPPL1 oligo corresponded to the BAR domain of human APPL1 (81–105 nucleotides): 5′-AAGAGTGGATCTGTACAATAA-3′ [26]. As a control, the sequence of the universal siRNA oligos was 5′-AUGAACGUGAAUUGCUCAATT-3′ (ST Pharm, Seoul, South Korea). The pEGFPC1-human APPL1 plasmid (plasmid #22198) was obtained from Addgene (Watertown, MA, USA), and the pHyPer-Cyto (HyPer-C) plasmid was purchased from Evrogen Joint Stock Company (Moscow, Russia). The full-length Rab5 sequence was amplified from the Rab5-CFP plasmid via PCR with the following primers to generate the early endosome-targetable HyPer (HyPer-Endo): forward, 5′-CCCAAGCTTATGGCTAGTCGAGGCGCAA-3′, and reverse, 5′-TTGGATCCTTAGTTACTACAACACTGATTC-3′. The amplified Rab5 coding region was cloned into the HindIII and BamHI sites of a stop codon-mutated HyPer-C vector (TAA→TTA). Rab5 was inserted at the C-terminal of a human catalase plasmid, whose C-terminal 12 nucleotides (encoding the KANL residues, a known peroxisome-targeting sequence) were removed, to construct the endosomal-targeting catalase (Cat-Endo) by using the same method as described above. The GFP-RhoB plasmid (plasmid #23225) was obtained from Clontech (Mountain View, CA, USA). CFP-Rab5 was kindly provided by Won Do Heo at KAIST (Daejeon, Republic of Korea).

### 2.4. Confocal Microscopy and Immunofluorescence

For live-cell imaging and immunofluorescence staining, cells were cultured on 12-well plates containing poly-L-lysine-coated coverslips (diameter 12 mm). For immunofluorescence (IF) staining, the cells were permeabilized using a solution containing 0.01% saponin (Sigma-Aldrich, St. Louis, MO, USA), 80 mM HEPES (pH 6.8), 5 mM EGTA, and 1 mM MgCl_2_ for 1 min to allow cytosolic leakage. Afterward, they were fixed with 4% formaldehyde in PBS on ice for 10 min. They were incubated with PBS containing 5% normal horse serum (Gibco, Grand Island, NY, USA) and 0.1% Triton X-100 at 25 °C for 30 min to block non-specific antibody binding. They were further incubated with rabbit or mouse primary antibodies (1:200 dilution) in a solution of 5% normal horse serum and 0.1% Triton X-100 in PBS for 30 min. They were quickly washed thrice with PBS and mixed with secondary antibodies (1:1000 dilution; Alexa Fluor 488 or 546 goat anti-rabbit or mouse IgG, Invitrogen, Carlsbad, CA, USA) in the same solution. For DNA staining, DAPI (0.2 μg/mL; Thermo Fisher Scientific, Waltham, MA, USA) was used. The samples were mounted onto glass slides with Fluoromount-G (Southern Biotech, Birmingham, AL, USA). Fluorescence images were captured under a confocal microscope (LSM 880 Airy, Carl Zeiss, Oberkochen, Germany, or Nikon A1R, Tokyo, Japan). Live cells were imaged using a Nikon A1R laser scanning confocal microscope equipped with a heated stage chamber (LCI, Seoul, Republic of Korea) and supplied with 5% CO_2_. Images were analyzed using the NIS-Elements AR 3.0 software (Nikon, Tokyo, Japan). The emission (Em_500_–_530nm_) intensity ratios of Ex_488nm_ vs. Ex_405nm_ were measured to assess the change in H_2_O_2_ levels in live cells expressing HyPer-Endo or HyPerC199S-Endo.

### 2.5. Immunoblot Analysis

Ice-cold PBS was used to wash the cells and stop the reactions before lysis. The cells (1 × 10^6^ in 100 mm dishes) were lysed in 0.5 mL of ice-cold lysis buffer containing 25 mM HEPES-NaOH (pH 7.0), 2 mM EDTA, 25 mM β-glycerophosphate, 1% Triton X-100, 10% glycerol, protease inhibitors (1 mM DTT, 5 mM NaF, 10 μg/mL aprotinin, and 10 μg/mL leupeptin), and a phosphatase inhibitor cocktail (Sigma-Aldrich, St. Louis, MO, USA). They were broken by sonication on ice for 2 min. Then, supernatants were collected via centrifugation at 12,000× *g* for 20 min for immunoblot analysis. Protein mixtures were resolved using 10% or 12% SDS-polyacrylamide gel electrophoresis at 100 V for 90 min. The separated proteins were transferred onto nitrocellulose membranes (Protran, Dassel, Germany) via electrophoresis at 600 mA for 120 min at 4 °C. The membranes were blocked and incubated overnight at 4 °C with primary antibodies diluted as follows: Rab5 (Cell Signaling Technology, 2143, rabbit, 1:1000), APPL1 (Cell Signaling Technology, 3858, rabbit, 1:1000), Akt (Cell Signaling Technology, 9272, rabbit, 1:1000), p-Akt(S^473^) (Cell Signaling Technology, 9271, rabbit, 1:1000), p-Akt(T^308^) (Cell Signaling Technology, 13038, rabbit, 1:1000), α-actin (Sigma-Aldrich, A3853, mouse, 1:1000), and GFP (Thermo Fisher Scientific, A11122, rabbit, 1:1000). Immune complexes were detected using horseradish peroxidase (HRP)-conjugated secondary antibodies in an ECL detection system with WESTSAVE Up reagents (Young In Frontier, Seoul, Republic of Korea). Band intensities were quantified using ImageJ 1.54a software (NIH, Bethesda, MD, USA).

### 2.6. Endocytosis Analysis

Cos7 cells were serum-starved for 5 h, washed with PBS twice, and incubated with DMEM containing Alexa Fluor 555-conjugated EGF (200 ng/mL; Thermo Fisher Scientific, Waltham, MA, USA) or Alexa Fluor 568-conjugated Transferrin (200 ng/mL; Thermo Fisher Scientific) in the presence or absence of glucose oxidase (20 mU; Sigma-Aldrich, St. Louis, MO, USA) at 37 °C for 30 min. They were briefly washed with cold PBS to terminate the reactions. Then, they were incubated with an acidic buffer (0.2 M acetic acid, 0.5 M NaCl) at room temperature for 5 min to remove fluorescent EGF or transferrin bound to the cell surface. Afterward, they were washed with cold PBS twice, fixed in 4% paraformaldehyde in PBS for 10 min, stained with DAPI to visualize the nuclei, and observed via confocal microscopy.

### 2.7. Statistical Analysis

All quantitative data were expressed as the means ± standard error of the mean (SEM) of multiple determinations from at least three independent experiments. Data were statistically analyzed using Student’s two-tailed *t*-test with Sigma Plot 10.0 (Systat Software, San Jose, CA, USA), and *p*-values were calculated to determine statistical significance.

## 3. Results

### 3.1. H_2_O_2_ Production in Early Endosomes During Epidermal Growth Factor (EGF) Activation Is Demonstrated via HyPer-Endo, an Endosomal-Targeting H_2_O_2_ Reporter

**Figure 1:**
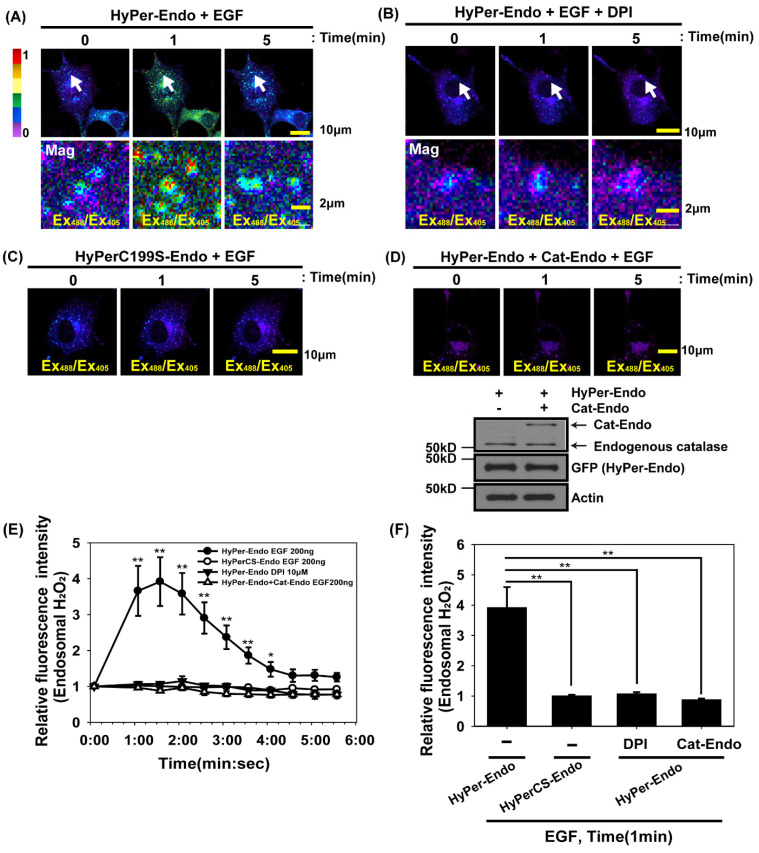
Increase in H_2_O_2_ levels in early endosomes in cells activated by growth factors. (**A**,**B**) Selected snapshot images of live HyPer-Endo-expressing Cos7 cells. The cells were incubated with EGF (200 ng/mL) for 1 or 5 min in the absence (**A**) or presence (**B**) of DPI (10 μM). DPI was preincubated for 20 min before the EGF treatment. The regions indicated by arrows are shown at a higher magnification in the second row. (**C**) Selected snapshot images of live Cos7 cells expressing HyPerC199S-Endo, a H_2_O_2_-insensitive mutant. The cells were treated with EGF as described above. (**D**) Selected snapshot images of live Cos7 cells co-expressing HyPer-Endo and Cat-Endo. The cells were activated by EGF. Protein expression was verified through immunoblot analysis by using antibodies against catalase and GFP to detect HyPer-Endo. The change in H_2_O_2_ levels in live cells was measured by the emission (Em_500–530nm_) intensity ratios of Ex_488nm_ vs. Ex_405nm_. (**E**,**F**) Relative fluorescence intensity (endosomal H_2_O_2_, baseline at time 0 to indicated times) was quantified in the cells treated as in (**A**–**D**). Data were shown as means ± SEM from three independent experiments (*n* = 11–14 cells per experiment). * *p* < 0.05, ** *p* < 0.01 (Student’s *t*-test).

To delineate the effect of regulation of Akt activity by H_2_O_2_ production on the endosomal complex, we transfected Cos7 cells with HyPer-Endo encoding a modified form of HyPer with a Rab5 small GTPase localized to the early endosomes (Figure S1A,B). The genetically encoded reversible H_2_O_2_ indicator HyPer can measure the dynamic change in H_2_O_2_ in cells by tracking the changes in the fluorescence properties of circularly permuted YFP (cpYFP, a rearranged version of YFP) resulting from the formation of an intramolecular disulfide bond between redox-sensitive Cys^199^ and Cys^208^ in the regulatory domain of OxyR in the presence of H_2_O_2_ [35,36]. Confocal immunofluorescence imaging revealed that HyPer-Endo was present near the EEA, a protein that identifies early endosomes (Figure S1B). HyPer-Endo visualized and quantified the dynamics of H_2_O_2_ in the early endosomes of the cells incubated with extracellular H_2_O_2_ (Figure S1C,E). Because the fluorescence of cpYFP in HyPer is also sensitive to changes in pH level [37], we produced the redox-insensitive and pH-sensitive vector HyPerC199S-Endo (HyPerCS-Endo), which encodes a modified form of HyPer-Endo in which Cys^199^ is replaced by Ser. The fluorescence of HyPerCS-Endo was not affected in the H_2_O_2_-treated cells (Figure S1D,E). Using HyPer-Endo and HyPerCS-Endo, we observed that early endosomal H_2_O_2_ levels transiently increased (Figure 1A,E,F), whereas the pH level in early endosomes (Figure 1C,E,F) in Cos7 cells did not change during EGF activation.

The treatment of DPI (a Nox inhibitor) and the expression of early endosome-targeting Cat-Endo, which is a modified form of catalase with a Rab5 small GTPase (Figure S2A), blocked endosomal H_2_O_2_ accumulation (Figure 1B,D–F). Confocal immunofluorescence imaging (Figure S2B) and a biochemical catalase assay from the lysates (Figure S2C) revealed that Cat-Endo was placed correctly in the endosomes and working properly. In previous studies using isolated endosomes from lysates of cells activated by cytokines such as TNF-α or interleukin-1, the increase in superoxide anion (O_2_^∙-^) was measured via electron paramagnetic resonance spectroscopy or lucigenin-based chemiluminescence [38,39]. Using HyPer-Endo, an endosome-targeting H_2_O_2_ sensor, we first visualized the increase in H_2_O_2_ levels around the early endosomes of the EGF-activated cells. Redoxosomes are potentially vital for transmitting cell signals from the plasma membrane to the cytosol in response to extracellular stimuli [5,40]. Given that H_2_O_2_ molecules act as local messengers, we next investigated whether redoxosomes were necessary for intracellular Akt signaling during EGF activation.

### 3.2. Akt Activation by H_2_O_2_ Production in Cells Activated by Growth Factors

**Figure 2:**
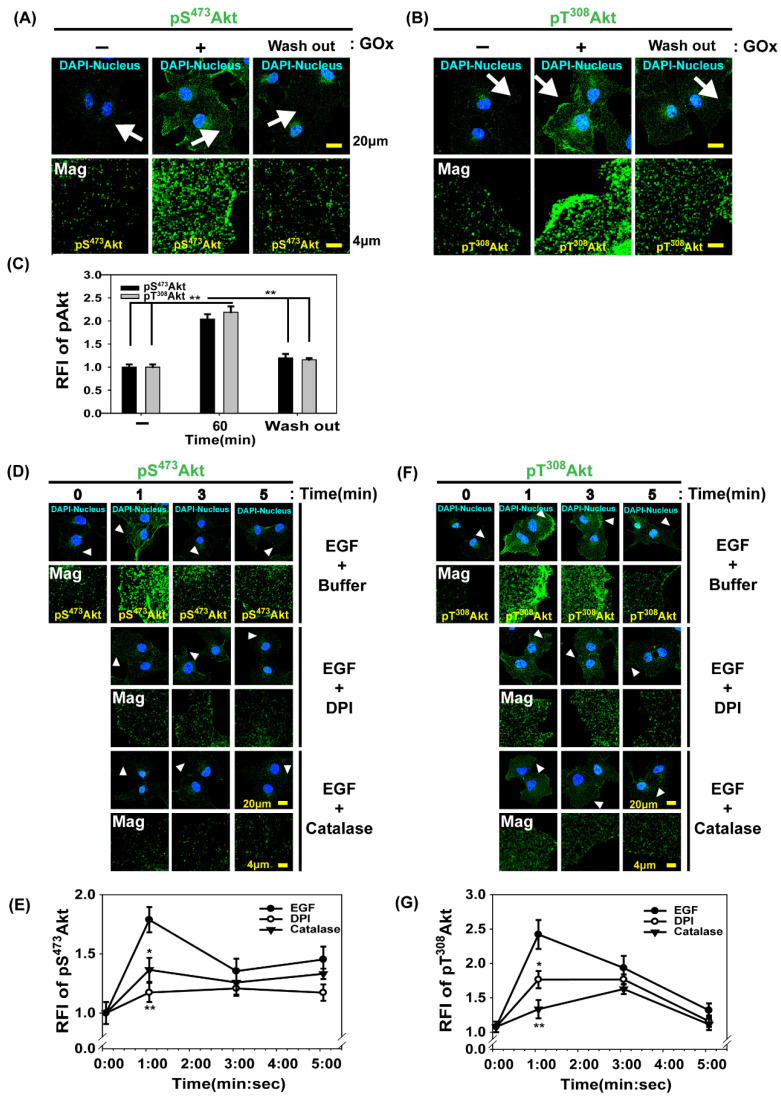
H_2_O_2_-mediated Akt activation via Ser^473^ phosphorylation and Thr^308^ phosphorylation in Cos7 cells during extracellular glucose oxidase treatment or EGF activation. (**A**,**B**) Cos7 cells were deprived of serum for 5 h and then incubated with GOx (20 mU/mL) in a high-glucose medium for 10 min. Alternatively, the cells were exposed to GOx for 10 min, washed, and incubated in the absence of GOx for an additional 1 h (wash out). The fixed cells were subjected to immunofluorescence analysis by using the antibodies to pS^473^Akt (**A**) or pT^308^Akt (**B**). The regions indicated by the arrows are shown at a higher magnification in the second row. The selected snapshot confocal microscopy images showed an increase in pS^473^Akt and pT^308^Akt levels. The regions indicated by the arrows are presented at a higher magnification. (**C**) The relative fluorescence intensities (RFI) of pS^473^Akt and pT^308^Akt are shown as means ± SEM (*n* = 4 for each condition). ** *p* < 0.01 (Student’s *t*-test). (**D**,**F**) Cos 7 cells were deprived of serum for 5 h, incubated in the absence (buffer) or presence of DPI (10 μM) for 30 min or catalase (2 mg/mL) as described in Section 2.2, and incubated with additional EGF (200 ng/mL) at the selected time points. The selected snapshot confocal microscopy images of fixed cells were shown for pS^473^Akt (**D**) and pT^308^Akt (**F**). Arrowheads indicate areas shown at a higher magnification. (**E**,**G**) The quantitative analysis of the RFI in (**D**,**F**) showed the phosphorylation levels of Akt at Ser^473^ and Thr^308^. Data are presented as means ± SEM (*n* = 7 cells for each condition). * *p* < 0.05, ** *p* < 0.01 (Student’s *t*-test) versus the corresponding values of cells with EGF and buffer for 1.0 min.

Thr^308^ in the catalytic core and Ser^473^ in a C-terminal hydrophobic motif on Akt1 [12] (corresponding Thr^309^ and Ser^474^ on Akt2; Thr^305^ and Ser^472^ on Akt3) should be phosphorylated to activate Akt kinase fully. The Thr^308^ phosphorylation of Akt1 by phosphoinositide-dependent protein kinase 1 (PDK1) is dependent on PtdIns(3,4,5)P_3_ production from PtdIns(4,5)P_2_ by PI3 kinase in the plasma membrane [41,42]. The Ser^473^ phosphorylation of Akt1 by the mechanistic target of rapamycin complex 2 (mTORC2) is controlled by PI3 kinase activation in cells treated with growth factors [13,43]; however, studies have yet to elucidate the mechanism by which m TORC2 kinase is recruited to the signaling complex containing Akt. Recently, we showed that in PDGF-activated glioma cells, endosomal phosphoinositide, which is represented by PtdIns(3,4)P_2_, is required for phosphorylation at Ser^473^ via mTORC2 [18]. To investigate how H_2_O_2_ molecules control intracellular Akt activation, we compared the changes in Thr^308^ or Ser^473^ phosphorylation levels in Akt between cells in the presence or absence of extracellular H_2_O_2_. Immunofluorescence confocal microscopy with the antibodies to phosphorylated Akt on Thr^308^ (pT^308^Akt) or Ser^473^ (pS^473^Akt) showed that the phosphorylation levels of Akt increased by approximately twofold at 10 min when Cos7 cells were exposed to a concentration of H_2_O_2_ constantly produced by GOx (20 mU/mL; Figure 2A–C). The pT^308^Akt or pS^473^Akt levels were reversed when the H_2_O_2_ source was removed. GOx (20 mU/mL) increased the extracellular H_2_O_2_ concentrations at a rate of approximately 1 μM/min during the oxidation of D-glucose to D-glucono-δ-lactone [44]. Next, we monitored Thr^308^ or Ser^473^ phosphorylation in Akt in Cos7 cells stimulated with EGF for 0, 1, 3, or 5 min by using antibodies to phosphorylate Akt and confocal microscopy (Figure 2D–G). Both Akt phosphorylation levels maximally increased 1 min after activation with EGF. The transient increases were blocked by DPI, indicating that H_2_O_2_ production was essential for Akt activation at early stages during EGF activation (Figure 2D–G). Confocal microscopy demonstrated that pS^473^Akt was widely distributed in a dotted pattern near the plasma membrane; conversely, pT^308^Akt was enriched in the plasma membrane. Considering that Akt should be sequentially phosphorylated on Thr^308^ and Ser^473^ to stimulate the kinase activity [12,13,45], we investigated the location of the spot pattern of pS^473^Akt in the cytosol. In a previous study, we showed that pS^473^Akt was present in early endosomes biochemically separated from cells activated by growth factors [18]. Therefore, a H_2_O_2_-dependent increase in pS^473^Akt levels in endosomes supports the theory that signaling endosomes provide the platform to create a connection between endocytosed activated receptors and cellular effectors for signal propagation [5,46,47,48].

### 3.3. Endosomal H_2_O_2_ Production Is Required for Akt Activation Through the Phosphorylation of Akt on Ser^473^

**Figure 3:**
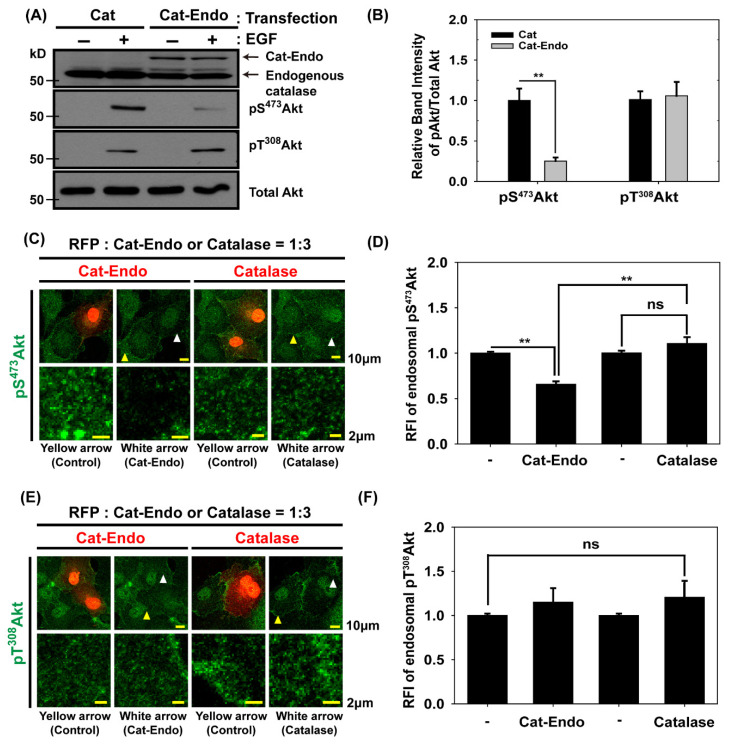
Selective decrease in Akt Ser^473^ phosphorylation by endosomal H_2_O_2_. (**A**) HeLa cells expressing catalase (Cat) or Cat-Endo were deprived of serum for 5 h and then treated with EGF (200 ng/mL) for 1 min. Cell lysates were then subjected to immunoblot analysis with the antibodies to the indicated proteins. (**B**) Akt phosphorylation levels at Ser^473^ and Thr^308^ residues from (**A**) were quantitatively evaluated. The relative immunoblot intensities of pS^473^Akt and pT^308^Akt normalized by those of the total Akt were determined as means ± SEM of three independent experiments. ** *p* < 0.01 (Student’s *t*-test). (**C**,**E**) Cos7 cells co-expressing the red fluorescent protein (RFP) and Cat-Endo or catalase were deprived of serum for 5 h and treated with EGF (200 ng/mL) for 1 min. The cells were transfected with the plasmids of pcDNA3-RFP and pCat-Endo or pCatalase at a ratio of 1:3 to select the cells expressing RFP and Cat-Endo or catalase simultaneously. They were permeabilized with 0.01% saponin-containing buffer to remove cytosolic signals. The fixed cells were subjected to immunofluorescence analysis by using antibodies to pS^473^Akt (**C**) or pT^308^Akt (**E**). Selected snapshot confocal microscopy images of pS^473^Akt (green), pT^308^Akt (green), and RFP (red). The regions indicated by the arrows are presented at a higher magnification. (**D**,**F**) The quantitative analysis of the relative fluorescence intensity (RFI) from (**C**,**E**) showed the Akt phosphorylation levels at Ser^473^ and Thr^308^. Data are presented as means ± SEM (four imaging sets for each condition). ** *p* < 0.01 (Student’s *t*-test) versus the corresponding values of the non-transfected cells.

To explore whether the forced reduction in H_2_O_2_ levels in early endosomes affected the phosphorylations of Akt on Ser^473^ and Thr^308^, we transfected HeLa cells with a plasmid encoding Cat-Endo with a Rab5 small GTPase; we then verified the Cat-Endo expression via an immunoblot analysis of the lysate from the transfected cells (Figure S2A). Immunofluorescence microscopy (Figure S2B) demonstrated that Cat-Endo was colocalized with GFP-RhoB, an endosomal marker protein [49]; this finding verified the presence of Cat-Endo in endosomes. The H_2_O_2_-degrading activity of Cat-Endo was comparable with that of wild-type catalase, as indicated by the Cat-Endo expression (Figure S2C). The immunoblots showed that the level of pS^473^Akt in Cat-Endo-expressing cells was reduced selectively by ~75% compared with that in catalase-expressing cells, but the pT^308^Akt level remained unchanged during EGF activation (Figure 3A,B). This result indicated that H_2_O_2_ production in endosomes was necessary to activate Akt through the accumulation of pS^473^Akt but not pT^308^Akt in EGF-activated Cos7 cells. Next, we investigated the cellular pS^473^Akt or pT^308^Akt levels between Cat-Endo-expressing cells with a relatively low endosomal H_2_O_2_ level and catalase-expressing control cells. Quantitative immunostaining revealed that endosomal pS^473^ Akt levels in Cat-Endo-expressing cells were ~40% lower than those in catalase-expressing cells; by comparison, endosomal pT^308^Akt levels between Cat-Endo- and catalase-expressing cells remained constant (Figure 3C–F). This result, together with immunoblot data (Figure 3A,B), demonstrated that Akt activation through the selective endosomal accumulation of pS^473^ Akt requires endosomal H_2_O_2_ production in EGF-activated cells. Since the Rab5 domain is present in the Cat-Endo protein, it is important to carefully examine whether the overexpression of the Rab5 domain impacts endosome function. Under our experimental setup, we did not detect any endosomal malfunction in Rab5-expressing cells.

### 3.4. APPL1, the Rab5 Effector and Akt Binding Protein, Is Localized in Early Endosomes Dependent on H_2_O_2_ Production

**Figure 4:**
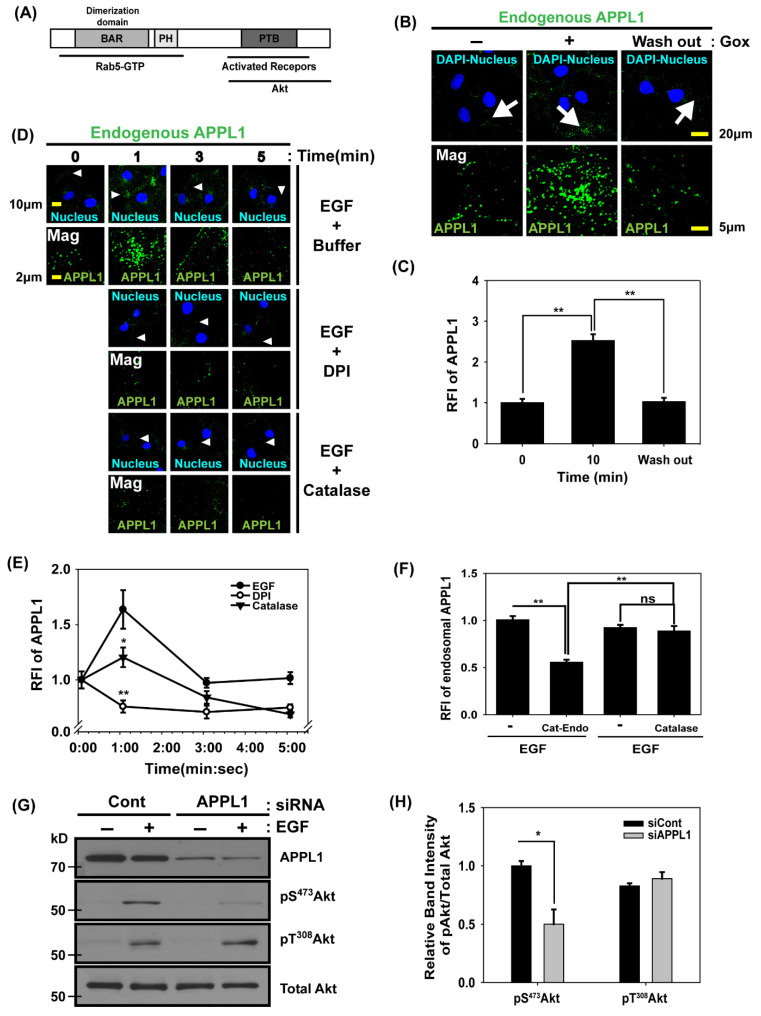
Movement of the Akt adaptor protein APPL1 from the cytosol to the early endosome by endosomal H_2_O_2_ during EGF activation. (**A**) Schematic representation of APPL1 domains showing three parts (gray boxes): BAR (essential for Rab5-GTP binding and dimerization), pleckstrin homology (PH, participating in association with Rab5-GTP), and phosphotyrosine binding (PTB, required for binding to activated receptors and Akt). (**B**) Cos7 cells were deprived of serum for 5 h and incubated in a high-glucose medium in the presence of GOx (20 mU/mL) for 10 min. Alternatively, the cells were exposed to GOx for 10 min, washed, and incubated in the absence of GOx for another 30 min (wash out). The fixed cells were subjected to immunofluorescence analysis by using the antibodies to APPL1. The regions indicated by the arrows are shown at a higher magnification in the second row. (**C**) Quantitative analysis of the relative fluorescence intensity (RFI) from (**B**). Data are presented as means ± SEM (*n* = 4 cells for each condition). ** *p* < 0.01. (**D**) Cos 7 cells were deprived of serum for 5 h, incubated in the absence (Buffer) or presence of DPI (10 μM) for 30 min or catalase (2 mg/mL), and incubated with additional EGF (200 ng/mL) at the selected time points. Selected snapshot confocal microscopy images of the fixed cells were shown for endogenous APPL1. Arrowheads indicate the areas shown at a higher magnification. (**E**) Quantitative analysis of the RFI from (**D**). Data are presented as means ± SEM (*n* = 7 cells for each condition). * *p* < 0.05, ** *p* < 0.01. (**F**) Cat-Endo- or catalase-expressing Cos7 cells were deprived of serum for 5 h and stimulated with EGF (200 ng/mL) for 1 min. The fixed cells were subjected to immunofluorescence analysis by using the antibodies to APPL1. The RFI of endosomal APPL1 is presented as means ± SEM (*n* = 3 images for each condition). ** *p* < 0.01. (**G**) HeLa cells were transfected with control siRNA (siCont) or siRNA for APPL1 (siAPPL1) for 72 h and deprived of serum for 5 h. After EGF (200 ng/mL) was added to the cells and left for 1 min, cell lysates were analyzed using antibodies against the proteins listed. (**H**) Quantitative analysis of the relative band intensity of pS^473^Akt and pT^308^Akt from (**G**). Data are presented as means ± SEM (*n* = 3 blots). * *p* < 0.05.

We examined the adaptor proteins for the recruitment of the Akt signaling complex into early endosomes. APPL1 is a Rab5 effector that interacts with Akt and endocytosed transmembrane receptors in endosomes. It contains an N-terminal Bar domain and a PH (pleckstrin homology) domain required for dimerization and endosomal localization; it also has a C-terminal PTB (phosphotyrosine binding) domain essential for binding to transmembrane receptors and Akt (Figure 4A) [28,50]. First, we examined the local change in APPL1 in cells under oxidative stress. Immunofluorescence confocal microscopy with APPL1 antibodies revealed that the extracellular GOx treatment induced an increase in endosomal APPL1 levels; furthermore, when the H_2_O_2_ source was removed, the increased APPL1 level was reversed (Figure 4B,C), indicating that H_2_O_2_ molecules enhanced the recruitment of APPL1 into endosomes. Next, we used the antibodies against APPL1 and confocal microscopy to measure the amount of endosomal APPL1 in Cos7 cells stimulated with EGF for 0, 1, 3, or 5 min. After the treatment with EGF, APPL1 in the endosomes increased by approximately 60% at 1 min compared with that before the treatment. The treatment with DPI or catalase protein blocked these transient increases. Therefore, H_2_O_2_ production was vital for the recruitment of APPL1 into endosomes at an early stage during EGF activation (Figure 4D,E). Next, we investigated the effect of endosomal H_2_O_2_ on APPL1 recruitment into early endosomes. The endosomal APPL1 levels in Cat-Endo-expressing cells were reduced by ~50% compared with those in the control cells during EGF activation (Figure 4F). We verified whether APPL1 affected Akt activation in the cells under EGF treatment. We compared the Akt phosphorylation levels between APPL1 knockdown cells and control cells during EGF activation. The immunoblots showed that the pS^473^ Akt level was reduced by ~50% in APPL1 knockdown cells compared with those in the control cells; however, the pT^308^Akt level remained unchanged (Figure 4G,H). Therefore, APPL1 is critical for the full activation of Akt through an increase in pS^473^ Akt levels.

We investigated the possibility that H_2_O_2_ molecules promoted receptor-mediated endocytosis and increased APPL1 recruitment into early endosomes. The endocytosis of EGF receptors and transferrin receptors remained consistent in cells during H_2_O_2_ production (Figure S3A–D). The number of Rab5-positive early endosomes did not change in the presence or absence of H_2_O_2_ (Figure S3E,F). This result showed that the number of APPL1-positive endosomes increased under oxidative stress because of changes in endosomal properties that caused APPL1 to be translocated to early endosomes rather than enhancing the receptor-mediated endocytosis.

### 3.5. Endosomal H_2_O_2_ via NADPH Oxidase (Nox) Complex Is Responsible for Akt Phosphorylation at S^473^ and mTORC2 Localization into Early Endosomes

**Figure 5:**
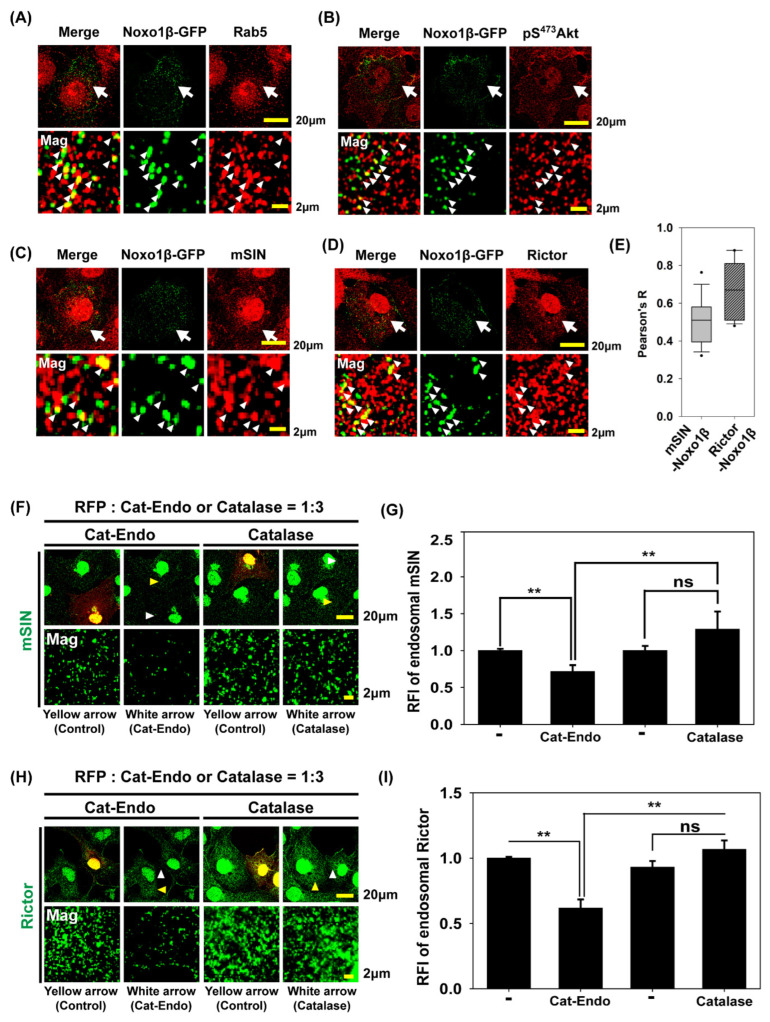
Enhancement of the recruitment of mTORC2, the kinase for Akt phosphorylation at Ser473, by endosomal H_2_O_2_ into early endosomes. (**A**–**D**) Cos7 cells expressing Noxo1β-GFP, a component of activated Nox1, were deprived of serum for 5 h and stimulated with EGF (200 ng/mL) for 1 min. The cells were permeabilized with 0.01% saponin-containing buffer to remove cytosolic signals. The fixed cells were subjected to immunofluorescence analysis by using the antibodies to Rab5 (**A**), pS^473^Akt (**B**), mSin (**C**), and Rictor (**D**). The regions indicated by the arrows are presented at a higher magnification. (**E**) Pearson correlation of mSIN and Noxo1β-GFP (**C**) or Rictor and Noxo1β-GFP (**D**) relative fluorescence intensity is shown in the graph. The correlation analysis is performed on the raw confocal images using the Coloc 2 plugin in ImageJ. Arrow-indicated endosomal puncta were manually selected as regions of interest (ROIs) for both mSIN1–Noxo1β and Rictor–Noxo1β panels (*n* = 19–21 areas). (**F**,**H**) Cos7 cells co-expressing the red fluorescent protein (RFP) and Cat-Endo or catalase were deprived of serum for 5 h and treated with EGF (200 ng/mL) for 1 min. The cells were transfected with the plasmids of pcDNA3-RFP and pCat-Endo or pCatalase at a ratio of 1:3 to select the cells expressing RFP and Cat-Endo or catalase simultaneously. They were permeabilized with 0.01% saponin-containing buffer to remove cytosolic signals. The fixed cells were subjected to immunofluorescence analysis by using the antibodies to mSIN (**F**) or Rictor (**H**). The selected snapshot confocal microscopy images are shown for mSIN (green), Rictor (green), and RFP (red). The region indicated by the white arrowhead is presented for the cells expressing Cat-Endo or catalase at a higher magnification. The region indicated by the yellow arrowhead is presented for the non-transfected control cell at a higher magnification. (**G**,**I**) The quantitative analysis of the relative fluorescence intensity (RFI) from (**F**,**H**) showed the relative endosomal mSIN and relative endosomal Rictor, respectively. Data are presented as means ± SEM (four imaging sets for each condition). ** *p* < 0.01 (Student’s *t*-test).

We examined whether the Nox complex was localized in endosomes during EGF stimulation. Since Nox1 is expressed in various cell types, and NADPH oxidase organizer 1 (Noxo1) is a critical component of Nox1 activation [51,52], we investigated the localization of activated Nox1 in Noxo1β-GFP-expressing Cos7 cells via confocal microscopy (Figure 5A–D). Noxo1β-GFP is localized in punctate vesicular structures and the plasma membrane. Rab5, a marker of early endosomes, colocalizes with a portion of Noxo1-GFP vesicles (Figure 5A). In endosomes, the part of pS473 Akt was also found with Noxo1β-GFP (Figure 5B). Two components, namely, mammalian Sty1/Spc1-interacting protein (mSIN) and rapamycin-insensitive companion of mTOR (Rictor) of mTORC2, a kinase in Akt phosphorylation at S^473^, are partially colocalized with Noxo1β-GFP (Figure 5C,D). The statistical colocalization analysis showed that the mean Pearson’s R value of mSIN-Noxo1β-GFP and Rictor-Noxo1β-GFP is 0.5 and 0.67, respectively, indicating moderate colocalization (Figure 5E). Next, we compared the amount of mSIN or Rictor in the endosomes between Cat-Endo- and catalase-expressing cells. Confocal microscopy analysis showed that the endosomal mSIN and Rictor levels in Cat-Endo-expressing cells were reduced by ~30% and ~40%, respectively, compared with those in the control cells; conversely, their levels remained consistent in the presence or absence of catalase overexpression (Figure 5F–I). These results indicated that the mTORC2 assembly in early endosomes required endosomal H_2_O_2_ production. Therefore, our data (Figure 4 and Figure 5) demonstrated that the endosomal recruitment of both Akt through APPL1 and mTORC2, a kinase for Akt, required endosomal H_2_O_2_ accumulation via Nox activation.

## 4. Discussion

In humans, Akt should be sequentially phosphorylated at Thr^308^ and Ser^473^ to fully activate Akt [12]. The selectivity of downstream substrate phosphorylation and the integrity of Akt signal transduction depend on the Akt phosphorylation state [45,53,54]. Previous reports showed that Akt is phosphorylated at Thr^308^ by PDK1 in the plasma membrane via the PtdIns(3,4,5)P_3_ accumulation and at Ser^473^ by mTorc2 via PtdIns(3,4,5)P_3_ or PtdIns(3,4)P_2_ accumulation in endosomes [13,18,55,56]. In cells stimulated with growth factors, H_2_O_2_ production in the plasma membrane via Nox inactivates PTEN through oxidation at the catalytic cysteine residue of this phosphatase; it also potentiates a transient increase in PtdIns (3,4,5)P_3_ levels to stimulate Akt signaling [15]. However, studies have yet to determine the role of intracellular H_2_O_2_ in Akt signal transduction. In the present study, we investigated the function of endosomal H_2_O_2_ molecules via Nox activation in terms of sequential Akt activation. We observed that the pS^473^ Akt levels were reduced in Cat-Endo-expressing cells compared with those in catalase-expressing cells. Together with the data showing an increase in H_2_O_2_ levels via the expression of HyPer-Endo, the results demonstrated that endosomes were critical for Akt signal transduction.

**Figure 6:**
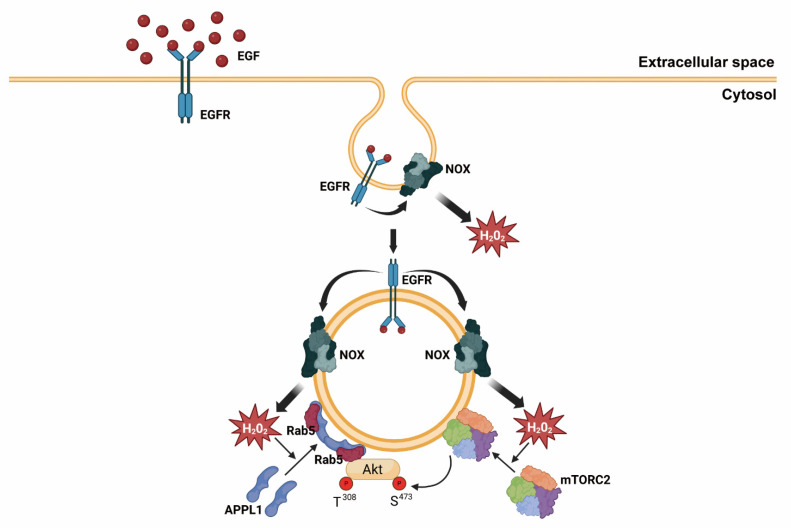
Proposed model illustrating the signaling role of endosomal H_2_O_2_ in Akt activation by the enhanced recruitment of APPL1 and mTorc2 in endosomes. See the Section 4 for details. EGFR, epidermal growth factor; NOX, NADPH oxidase.

Figure 6 shows our model that illustrates the mechanism by which H_2_O_2_ accumulates in early endosomes and its regulatory role in Akt signaling. An activated EGF receptor induces H_2_O_2_ production via the activated Nox at the plasma membrane. H_2_O_2_ molecules at the plasma membrane then oxidize target proteins containing redox-sensitive cysteine, including protein tyrosine phosphatases (PTPs) and the tumor suppressor PTEN; thus, they transduce signals through the accumulation of tyrosine phosphorylated proteins or PtdIns(3,4,5)P_3_, a docking platform of PDK1 and Akt [57,58]. Early endosomes are formed at EGFR- and Nox-containing plasma membrane enriched with PtdIns(4,5)P_2_ and PtdIns(3,4)P_2_. Increased H_2_O_2_ molecules near endosomes have two roles in Akt signaling. Endosomal H_2_O_2_ promotes the transport of Akt into the endosome from the cytosol by recruiting APPL1. Simultaneously, mTORC2 is transported to endosomes by H_2_O_2_; then, it phosphorylates the neighboring Akt at Ser^473^. When Akt is fully activated and phosphorylated at Thr^308^ and Ser^473^, it increases the phosphorylation levels of GSK3β and FoxO1/3a, which sends specific signals. The presence of pS^473^ Akt in early endosomes of EGF-activated cells is similar to our previous data, which showed that pS^473^ Akt levels increase in early endosomes in PDGF-activated cells [18]. In cells responding to various stimulations, including EGF (in this study), PDGF [18], and insulin-like growth factor-1 (IGF-1) [28], Akt is localized and activated in early endosomes. Therefore, early endosomes have a preserved role in Akt signal amplification and fidelity.

H_2_O_2_, an incompletely reduced oxygen metabolite, elicits various physiological and pathological effects on living cells depending on the amount, time, and location of its generation. As a well-known intracellular messenger, it regulates the intracellular signaling pool by activating PI3K and its downstream target, Akt, in mammalian cells activated by various stimuli [15,57]. Given that H_2_O_2_ molecules at concentrations above 10–100 μM are toxic to cells [59], Nox-derived H_2_O_2_ concentration increases rapidly, remains sufficiently high to oxidize target molecules, and reaches a low intracellular level because of H_2_O_2_-eliminating peroxiredoxins (Prxs), which are abundant in cells [57]. The localized PrxI inactivation through phosphorylation at Tyr^194^ and PrxII by hyperoxidation allows receptor-activated signaling to maintain and transduce into the cytosol [8]. Increased H_2_O_2_ levels in the plasma membrane are used as a mediator of cell signal propagation. H_2_O_2_ oxidizes PTPs to transduce the signal of protein tyrosine phosphorylation into the cytosol [58] and inactivates PTEN lipid-3 phosphatase to transduce the PI3K signal by accumulating PtdIns(3,4,5)P_3_ [15]. It also oxidizes synaptojanin lipid dual phosphatase to increase PtdIns(4)P and PtdIns(3,4)P_2_ during receptor-mediated endocytosis [60] in mammalian cells in response to various extracellular stimuli, including EGF and PDGF. Several studies have shown endosomal Nox-derived H_2_O_2_ production [5,7,61], but studies have yet to explore the consequence of its local enhancement in response to EGF. In the present study, endosomal Akt activation through the increased pS^473^Akt depended on endosomal H_2_O_2_ accumulation, as demonstrated by the reduced levels of pS^473^Akt, mTORC2, and APPL1 in cells expressing early endosome-targeting catalase (Cat-Endo) compared with those in cells expressing catalase.

## 5. Conclusions

In this study, we show that the increased concentration of endosomal H_2_O_2_ molecules enhances the recruitment of APPL1 Akt adaptor protein and mTORC2 Akt kinase to early endosomes during growth factor activation. Thereafter, Akt becomes fully active through Ser^473^ phosphorylation by mTORC2 in early endosomes. This Akt activation induced by endosomal H_2_O_2_, together with plasma membrane H_2_O_2_-dependent Akt activation through Thr^308^ phosphorylation by PDK1, contributes to the specificity and fidelity of Akt signaling. Redoxosomes were proposed to have a relatively high ROS level [5]. Thus, consistent with previous studies [4,5,8,34,62,63], the present study demonstrates the physiological effect of redoxosome on Akt signal transduction and supports the role of locally accumulated intracellular H_2_O_2_ as signaling mediators around organelles and biomolecular condensates.

## Data Availability

All the data discussed in the paper will be accessible to the readers. The raw datasets are available from the corresponding author upon reasonable request.

## Supplementary Materials

The following supporting information can be downloaded at https://www.mdpi.com/article/10.3390/antiox14050594/s1: Figure S1: HyPer-Endo detects endosomal H_2_O_2_ accumulation in a redox-sensitive cysteine-dependent manner; Figure S2: Intracellular localization and activity of Cat-Endo, an endosome-targeting catalase; and Figure S3: Receptor-mediated endocytosis remained unchanged in cells with extracellular H_2_O_2_ production at a rate of roughly 1 μM/min.

## Abbreviations

APPL1	Adaptor protein containing PH domain, PTB domain, and Leucine zipper motif 1
CFP	Cyan fluorescent protein
DPI	Diphenyleneiodonium chloride
EEA1	Early endosome antigen 1
EGF	Epidermal growth factor
FOXO1/3	Forkhead box O1/O3
GFP	Green fluorescent protein
GOx	Glucose oxidase
GSK 3	Glycogen synthase kinase 3
H_2_O_2_	Hydrogen peroxide
IGF	Insulin-like growth factor
mSIN	Mammalian Sty1/Spc1-interacting protein
mTORC2	Mechanistic target of rapamycin (mTor) complex 2
Nox	NADPH oxidase
Noxo1	NADPH oxidase organizer 1
PDGF	Platelet-derived growth factor
PDK1	Phosphoinositide-dependent protein kinase 1
PI3K	Phosphoinositide 3-kinase
Prx	Peroxiredoxin
PtdIns	Phosphatidylinositol
PTEN	Phosphatase and tensin homolog
PTP	Protein tyrosine phosphatase
Redoxosome	Redox-active endosome
Rictor	Rapamycin-insensitive companion of mTOR
RME	Receptor-mediated endocytosis
ROS	Reactive oxygen species
SHIP	The SH2-containing inositol 5′-phosphatase
TSC2	Tuberous sclerosis 2
