# Supplementary material for: Endosomal H2O2 Molecules Act as Signaling Mediators in Akt/PKB Activation
Source: Antioxidants (Basel). 2025 May 16;14(5):594. doi: 10.3390/antiox14050594 (PMC12108365; doi:10.3390/antiox14050594)
Supplement: Supplementary file 1 [file antioxidants-14-00594-s001.zip › antioxidants-3610606-supplementary.pdf]

Article for Antioxidants

Endosomal H_2O_2 molecules act as signaling mediators in Akt/PKB activation

Sujin Park[1,†], Chaewon Kim[1,†], Sukyeong Heo[1,2], and Dongmin Kang[1,*]

[1]Department of Life Science, Fluorescence Core Imaging Center and Bioimaging Data Curation Center, Ewha Womans University, Seoul 03760, Republic of Korea
[2]Department of Biomedical Engineering, Dongguk University, Seoul, 10326, Republic of Korea

Abbreviated title: Akt/PKB activation by endosomal H_2O_2

[*] Correspondence: dkang@ewha.ac.kr

†These authors contributed equally to this work.

Keywords:
Receptor-mediated endocytosis, hydrogen peroxide, Akt/PKB, early endosome, APPL1

Supplementary Fig. S1

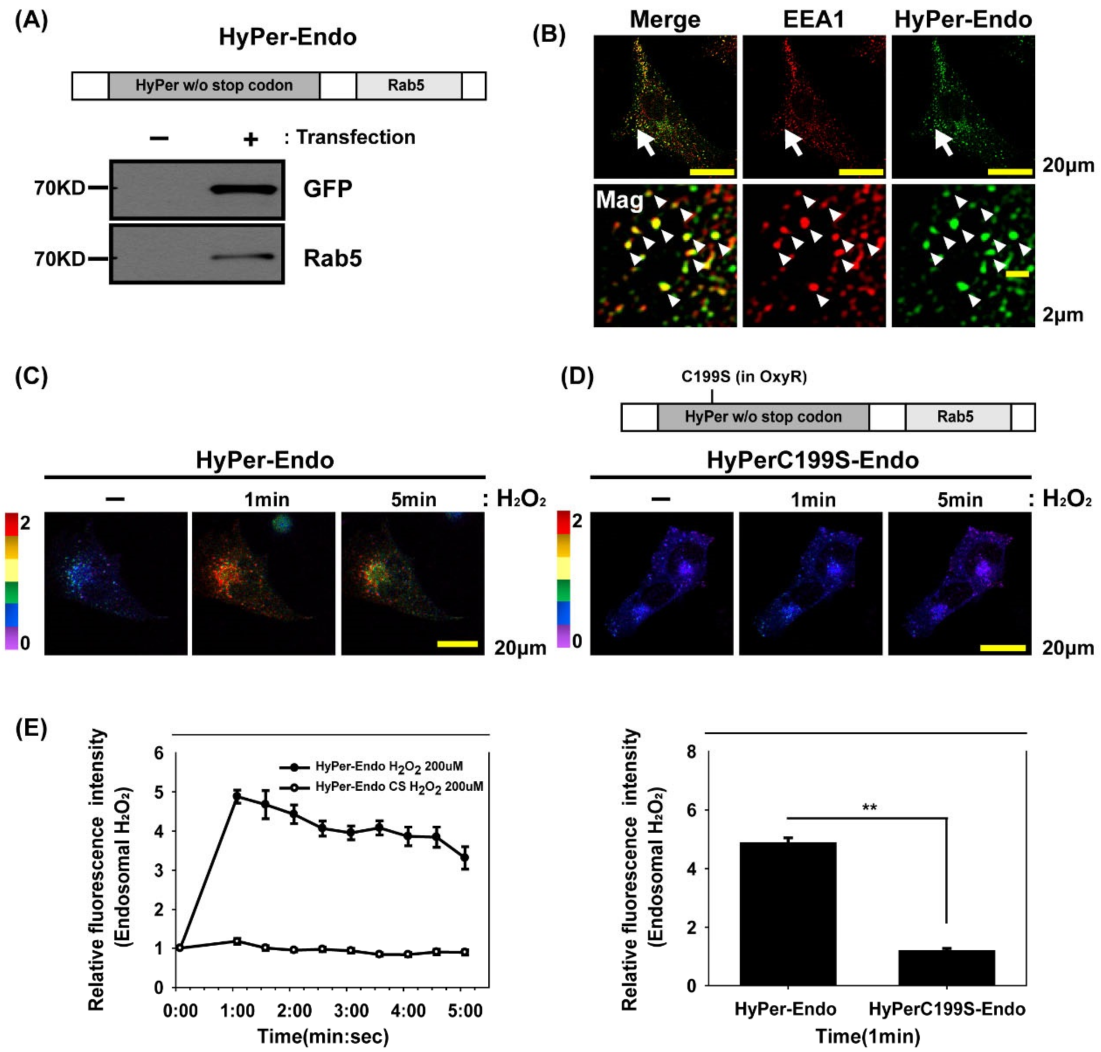

Supplementary Figure S1. HyPer-Endo detects endosomal H_2O_2 accumulation in a redox-sensitive cysteine-dependent manner. (**A**) Schematic representation of HyPer-Endo (in which the early endosome-targeting Rab5 sequence is conjugated at the C-terminus site of HyPer). Western blot analysis of the lysates from the transfected Cos7 cells was performed to confirm their protein expression. (**B**) HyPer-Endo-expressing Cos7 cells were subjected to immunofluorescence confocal microscopy with antibodies to EEA1 (red, early endosome) and GFP (green, HyPer-Endo). The area indicated by the arrows was shown at a higher magnification in the lower row. Arrowheads indicate the colocalized regions between HyPer-Endo and endogenous EEA1. (**C,D**) HeLa cells expressing HyPer-Endo (**C**) and HyPerC199S-Endo (**D**) were incubated with extracellular H_2O_2 (200 μM) for confocal live cell imaging. The selected snapshot images were shown at the indicated time points. (**E**) Quantification of relative endosomal fluorescence in (**C,D**). H_2O_2 levels were measured using relative emission activated by 488 and 405 nm lasers (Em_{488}/Em_{405}). The bar graph shows the relative fluorescence intensity at 1 min in H_2O_2-treated HeLa cells. Data were presented as means ± SEM (n = 7). **$P < 0.01$.

Supplementary Fig. S2

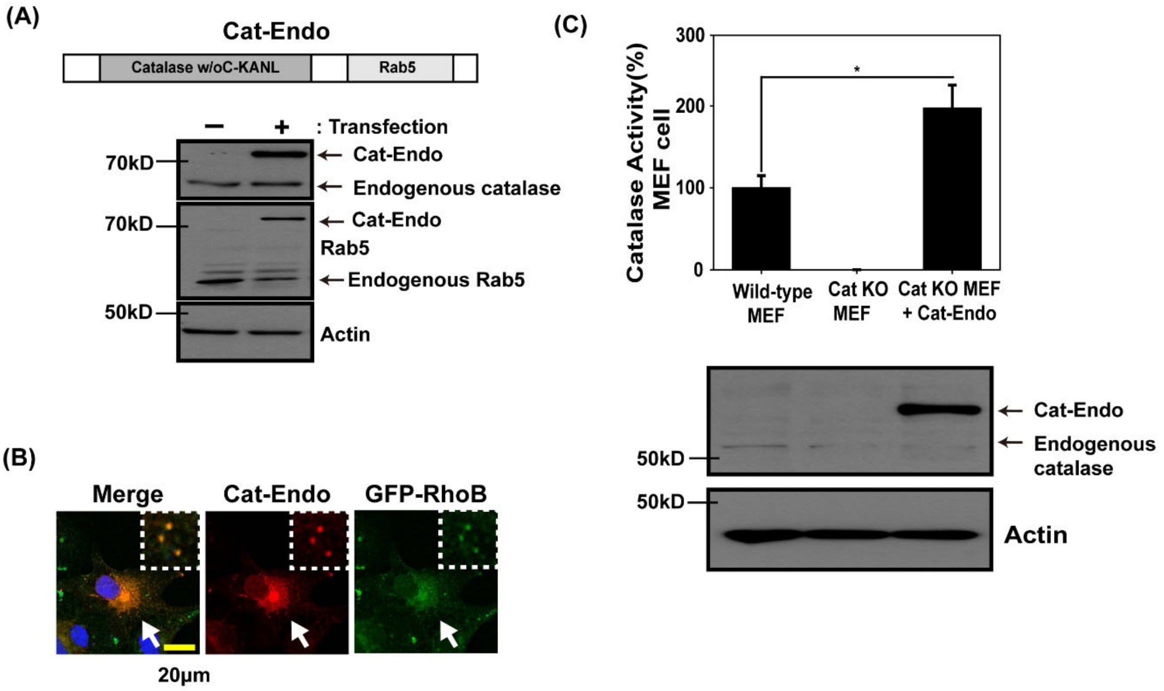

Supplementary Figure S2. Intracellular localization and activity of Cat-Endo, an endosome-targeting catalase. (**A**) Schematic representation of an endosome-targeting catalase (Cat-Endo). Immunoblot analysis of Cat-Endo-expressing Cos7 cells (molecular weight = approximately 85 kDa) was performed on the lysates to verify their expression by using antibodies to catalase and Rab5. (**B**) The localization of Cat-Endo in the endosome was assessed in Cos7 cells co-expressing Cat-Endo (red) and GFP-RhoB (green) via confocal microscopy. The immunofluorescence analysis of Cat-Endo-expressing cells was performed with antibodies to catalase. DNA was stained with DAPI (blue). The area indicated by the arrows (endosomes) is shown at a higher magnification in each inset. GFP-RhoB was used as an endosome marker. (**C**) Cat-Endo activity was analyzed using the Amplex Red assay kit (Molecular Probes). Wild-type (WT) mouse embryonic fibroblast (MEF) cells and catalase knockout (KO) MEF cells were used. Neon electroporation at a final concentration of 10 μg/mL plasmid per reaction was conducted to express Cat-Endo in catalase KO MEF cells. The bar graph shows the relative catalase activity from the lysates. Data were presented as means ± SEM ($n = 3$). *$P < 0.05$. The immunoblot from the lysates with the antibodies to catalase revealed the Cat-Endo expression.

Supplementary Fig. S3

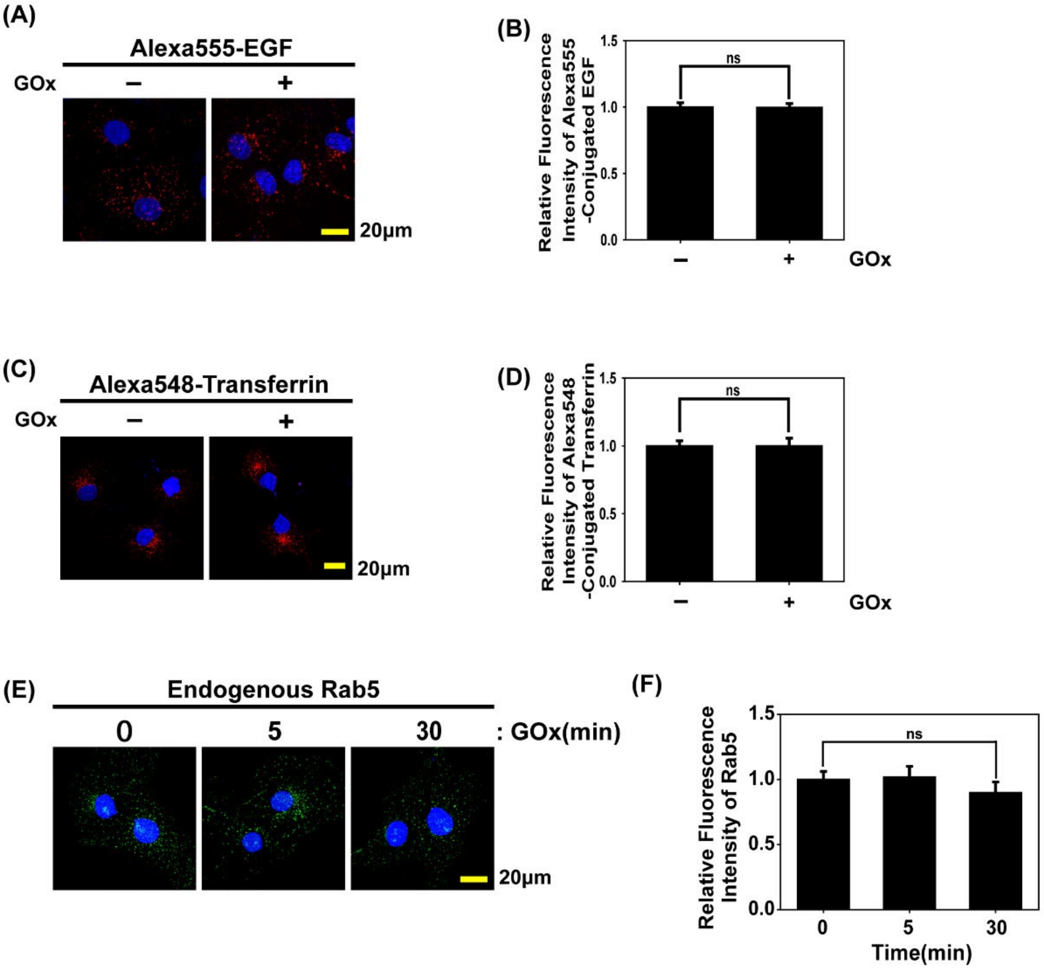

Supplementary Figure S3. Receptor-mediated endocytosis remained unchanged in cells with extracellular H_2O_2 production at a rate of roughly 1 μM/min. (**A,C**) Cos7 cells were deprived of serum for 5 h and incubated with Alexa Fluor 555-conjugated EGF (200 ng/mL) (**A**) or Alexa Fluor 546-conjugated transferrin (200 ng/mL) (**B**) in the presence or absence of glucose oxidase (20 mU/mL) for 30 min. Confocal microscopy revealed the representative snapshot images of endocytosed EGF (red) and transferrin (red). (**B.D**) The relative fluorescence intensities in (**A**) and (**C**) was quantified. Data were presented as means ± SEM (three imaging sets for each condition). (**E**) Cos7 cells were deprived of serum for 5 h and incubated with glucose oxidase (20 mU/mL) for 5 or 30 min. They were permeabilized with 0.01% saponin-containing buffer to remove cytosolic signals. The fixed cells were subjected to immunofluorescence analysis by using antibodies to Rab5. Representative snapshot confocal microscopy images of endosomal Rab5 (green) are shown. (**F**) Quantitative analysis of the relative fluorescence intensity in (**E**). Data were presented as means ± SEM (three imaging sets for each condition).
